# Recovery from Eating Disorders: A Guide for Clinicians and Their Clients

**DOI:** 10.1192/pb.bp.113.044933

**Published:** 2014-06

**Authors:** Ulrike Schmidt

Eating disorders in general - and anorexia nervosa in particular - are bewildering and frightening for families, clinicians and patients alike. They are bewildering because in contrast to any other psychiatric disorder, patients often value at least some aspects of their disorder (e.g. being able to restrict their food intake, extreme exercise) and are reluctant to change. These disorders are frightening because of the often dramatic physical consequences and high medical risks involved. Patients and their families alike often feel extremely alone and isolated.

This is a unique book: it is based on more than 100 in-depth interviews with people with eating disorders conducted by the author. The book’s authority derives in part from the many different voices of people with eating disorders, reflecting on different stages of their illness and illustrated by evocative quotes and in part from the wise, nuanced and straightforward commentary of its author, which gives context to these quotes.

The structure of the book follows the journey into and out of the illness, describing vividly the typical antecedents and common trajectories into an eating disorder, with classical tipping points into the disorder and the often arduous journey of turning things around towards recovery.

The lure and positives of the illness and treacherous friendship it provides is explored as much as the downside, as are the motivations for change and recovery.

The book’s core focus on the lived experience of people with eating disorders will be very informative for patients and families alike, and is useful ‘food for thought’ even for seasoned clinicians trying to get a fresh view on how to help their patients turn things around.

Multiple helpful questionnaires and checklists are provided that can be used by patients as self-assessment tools either in a self-help or therapeutic context. Although the book is full of useful information, advice and clinical wisdom, it never lectures or tries to persuade; rather, it lets former patients speak of their journey and what was helpful. As such, this book is both hopeful and realistic.

